# The application of the fibroblast activation protein α-targeted immunotherapy strategy

**DOI:** 10.18632/oncotarget.8098

**Published:** 2016-03-15

**Authors:** Guan-Min Jiang, Wei Xu, Jun Du, Kun-Shui Zhang, Qiu-Gui Zhang, Xiao-Wei Wang, Zhi-Gang Liu, Shuang-Quan Liu, Wan-Ying Xie, Hui-Fang Liu, Jing-Shi Liu, Bai-Ping Wu

**Affiliations:** ^1^ Department of Clinical Laboratory, Hunan Cancer Hospital and The Affiliated Cancer Hospital of Xiangya School of Medicine, Central South University, Changsha, Hunan, China; ^2^ Department of Clinical Laboratory, The First Affiliated Hospital of University of South China, Hengyang, Hunan, China; ^3^ Department of Microbial and Biochemical Pharmacy School of Pharmaceutical Sciences, Sun Yat-sen University, Guangzhou, Guangdong, China; ^4^ Department of Pharmacy, The Second Affiliated Hospital of Sun Yat-sen University, Guangzhou, Guangdong, China; ^5^ Department of Radiation Oncology, Hunan Cancer Hospital and The Affiliated Cancer Hospital of Xiangya School of Medicine, Central South University, Changsha, Hunan, China; ^6^ Department of Anesthesia, Hunan Cancer Hospital and The Affiliated Cancer Hospital of Xiangya School of Medicine, Central South University, Changsha, Hunan, China

**Keywords:** fibroblast activation protein α, tumor microenvironment, immune suppression, immunotherapy

## Abstract

Cancer immunotherapy has primarily been focused on attacking tumor cells. However, given the close interaction between tumor cells and cancer-associated fibroblasts (CAFs) in the tumor microenvironment (TME), CAF-targeted strategies could also contribute to an integrated cancer immunotherapy. Fibroblast activation protein α (FAP α) is not detectible in normal tissues, but is overexpressed by CAFs and is the predominant component of the stroma in most types of cancer. FAP α has both dipeptidyl peptidase and endopeptidase activities, cleaving substrates at a post-proline bond. When all FAP α-expressing cells (stromal and cancerous) are destroyed, tumors rapidly die. Furthermore, a FAP α antibody, FAP α vaccine, and modified vaccine all inhibit tumor growth and prolong survival in mouse models, suggesting FAP α is an adaptive tumor-associated antigen. This review highlights the role of FAP α in tumor development, explores the relationship between FAP α and immune suppression in the TME, and discusses FAP α as a potential immunotherapeutic target.

## INTRODUCTION

In the past, cancer immunotherapy mainly focused on attacking tumor cells. Cancer immunotherapy is a promising treatment strategy against solid tumors; however, it cannot completely eradicate them. The biological complexity of the tumor microenvironment (TME) seems to be an obstacle for cancer immunotherapy, suggesting that using a strategy in which only tumor cells are targeted is inadequate to overwhelm the aggressively growing tumor. Therefore, a synergistic TME-targeted strategy is required for the development of more potent cancer immunotherapy [1]. Modern immunotherapy has shifted to an approach that also targets the TME. This approach is founded on the “seed and soil hypothesis” which has illuminated that the complex interplay between TME components plays an important role in tumor metastasis [2]. Among tumor stromal cell types, cancer-associated fibroblasts (CAFs) are the dominant cellular component in the TME, and they play critical roles in promoting tumor progression. Given the close interaction between tumor cells and CAFs in the TME, CAF-targeted strategies would be promising for developing integrated cancer immunotherapy [3]. Fibroblast activation protein α (FAP α) is overexpressed by CAFs and is the predominant component of the stroma in most types of cancer, while it is not detectable in normal adult human tissues [4, 5]. Studies have confirmed that FAP α plays multiple roles in neoangiogenesis, invasion, and metastasis; thus, FAP α has been explored as a target for cancer therapy. Combined immunotherapy treatment with T cells that target cancer cells and an additional agent that targets FAP α-expressing cells for destruction could increase the success of solid tumor elimination [6, 7].

In order to determine the role of FAP α-expressing stromal cells in immune suppression within the TME, a transgenic mouse model has been created with established Lewis lung carcinomas. Only 2% of the tumor cells in this model express FAP α; however, when all FAP α expressing cells (stromal and cancerous) are destroyed, the tumors begin to die rapidly. Therefore, FAP α expressing cells are a non-redundant, immunosuppressive component of the TME [8]. Furthermore, it was reported that a FAP α vaccine combined with curcumin stimulates FAP α antibody production and CD8+ T cell-mediated killing of FAP α-expressing stromal cells and prolongs the survival of mice implanted with melanoma [9]. All of these results suggest that FAP α is an adaptive tumor-associated antigen useful for tumor immunotherapy. In this article, we will review the role of FAP α in tumor development, discuss FAP α as a potential target, and examine its immunotherapeutic benefits.

## THE BIOLOGICAL CHARACTERISTICS OF FIBROBLAST ACTIVATION PROTEIN α

Extracellular matrix (ECM) breakdown, detachment of neoplastic cells from the primary site, and their subsequent invasion into the lymphatic vessels, capillaries, and the surrounding normal tissues, is the result of a complex interplay of numerous proteolytic enzymes, including serine proteases. Overexpression of serine proteases in carcinomas is correlated with enhanced tumorigenicity and adverse prognosis [10-12]. Therapeutic strategies targeting FAP α, a membrane-bound serine protease of the prolyl oligopeptidase family that is expressed on CAFs within the tumor stroma, offer another tumor treatment option [13].

FAP α was originally identified by a group in pursuit of a selective marker for activated fibroblasts. A monoclonal antibody named F19 was produced to define the FAP α-positive cell, which strongly labeled cultured fibroblasts, fibroblasts in fetal mesenchymal tissues, the reactive stromal fibroblasts of epithelial tumors, and tumor cells of sarcomas [5, 14]. The name FAP α was given to the F19 antigen because of its unique expression profile [15]. Subsequently, another group identified a 170-KDa membrane-bound protease expressed by invadopodia, the protrusions of invasive melanoma cells, which was called “seprase”[16, 17]. Molecular cloning suggested that FAP α and seprase were the same cell surface serine protease [18-20].

**Figure 1:**
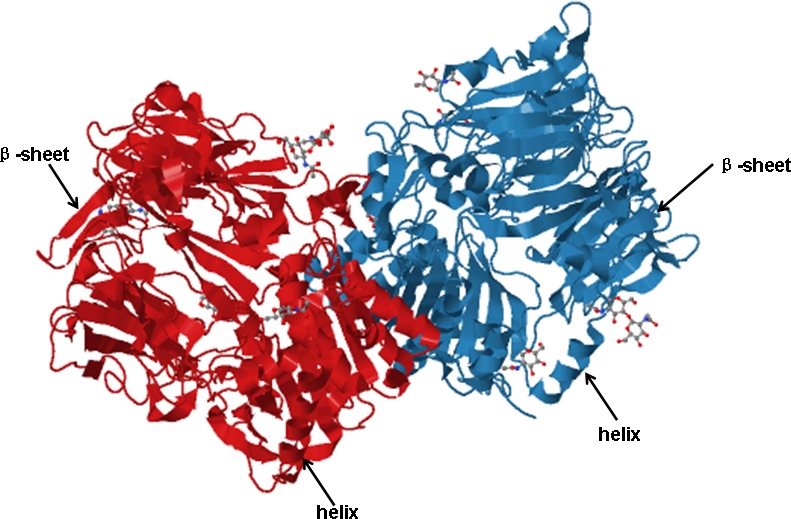
Cartoon architecture of the FAP α homodimer The critical structure of the catalytic triad is formed by serine (Ser624), aspartate (Asp702), and histidine (His734), and Ser624 is essential for enzymatic activity. The figure was generated using JSmol (PDB ID 1Z68). The blue and red represents two same subunit of FAP α which contains helixes and β-sheets.

FAP α is a type II integral membrane serine protease that belongs to the dipeptidyl peptidase (DPP) subfamily, which has the ability to cleave the bond between proline and any other amino acids. This enzymatic activity has been shown to have an impact on a wide variety of bioactive signaling molecules [21]. There is 50% sequence homology between DPP-IV and FAP α, and 70% homology in the catalytic domain. Both peptides have the same domain structure and belong to the family of post-prolyl peptidases [22, 23]. Human FAP α, expressed in activated stromal fibroblasts and remodeling tissue, is a type II cell-surface-bound transmembrane glycoprotein with Mr 95,000, it consists of 760 amino acids and is composed of a short 6 amino acid cytoplasmic domain, an 18 amino acid trans-membrane domain, and a large extracellular domain of 736 amino acids. The critical structure of the catalytic triad is formed by serine (Ser624), aspartate (Asp702), and histidine (His734) [24] (Figure 1). Ser624 is essential for enzymatic activity, for when this serine is changed into alanine, the proteolytic activity of FAP α no longer exists [25]. According to the crystal structure, FAP α exists as a homodimer which, when activated, must assemble into a heterodimer [26]. FAP heterodimers which composed by FAP α and FAP β participate in the migration of fibroblasts to collagen substrates [27], probably because together they can effectively degrade the substrate and regulate tumor cell growth, differentiation, adhesion, metastasis.

FAP α has both dipeptidyl peptidase and collagenase activity, and can degrade gelatin and type I collagen. However, the biological significance of FAP α cleavage of gelatin and type I collagen is still unknown. Nonetheless, FAP α is required for the generation of biologically active fragments of denatured collagen [28]. Recently, Neuropeptide Y (NPY), B-type natriuretic peptide, substance P, and peptide YY were found to be natural substrates for FAP α dipeptidyl peptidase activity. These proteins are also substrates for DPPIV; however, FAP α is distinguished from DPPIV by its effects on the half-life of substrates and its endopeptidase activity [29, 30].

## THE ASSOCIATION BETWEEN FIBROBLAST ACTIVATION PROTEIN α AND HUMAN CARCINOMA

While different kinds of tumors have general features in common, they also have their own characteristics related to tumor location, size, stage, degree of cell malignancy, involvement of lymph nodes, and metastasis. In this section, we will examine the role of FAP α in different carcinomas. FAP α is expressed by more than 90% of human epithelial tumors, and there has been much interest in exploring FAP α as a therapeutic target in breast cancer. *In vivo* studies have demonstrated that increased FAP α expression is associated with increased tumor growth rate and promotes neovascularization [31]. Another study using shRNA to target FAP α in a mouse model carrying 4T1 breast cancer came to the same conclusions [32]. These two results demonstrate the important role of FAP α and its potential value as an effective therapeutic target.

FAP α is overexpressed by CAFs in 85-90% of primary and metastatic colorectal cancers [33]. High levels of FAP α in human colon tumors promote tumor growth, progression, metastasis, and recurrence [34]. Moreover, the level of FAP α in rectal carcinomas, which have received preoperative chemo- or radiotherapy, is a negative prognostic factor [35]. Not only the level of FAP α, but also the location of FAP α, is related to poor prognosis of colon cancer patients [33]. All of these findings provide rationale for the development of FAP α-directed therapy.

A series of findings about the expression and role of FAP α in pancreatic carcinoma has suggested that FAP α-targeted immunotherapy may be a new treatment for pancreatic cancer patients. FAP α-induced reorganization of the ECM in TME promotes the invasiveness of pancreatic cancer cells [36]. There is also growing evidence that high FAP α expression in pancreatic cancer is related to poor clinical outcome and its location is associated with its clinical results [37]. In pancreatic carcinoma, FAP α is not only expressed in stromal fibroblast cells, but also in carcinoma cells, in contrast to previous studies which had shown FAP α to be selectively expressed in malignant cells of bone and soft tissue sarcomas. In addition, similar to previous findings, high expression of FAP α in fibroblasts and carcinoma cells is associated with poor clinical outcomes. Therefore, FAP α is a link between the TME and pancreatic cancer cells, which indicates that blocking the activity of FAP α directly or depleting the FAP α-expressing cells may obtain the expected anti-tumor effects [38]. Although the exact function of FAP α in the development of the different diseases remains unclear, it is believed to participate in the progression and metastasis of cancer, angiogenesis, and the suppression of the antitumor response of the immune system [4]. In sum, these findings support the hypothesis that FAP α is a novel target for tumor therapy.

## THE RELATIONSHIP BETWEEN FIBROBLAST ACTIVATION PROTEIN α AND IMMUNE SUPPRESSION IN THE TUMOR MICROENVIRONMENT

The complex interactions between the stroma and tumor, along with the regulatory signaling molecules in the TME, contribute to oncogenesis and tumor progression. The process of tumor invasion and metastasis is accompanied by angiogenesis and ECM degradation [39]. In most epithelial cancers greater than 1-2mm^3^ in size, tumor progression is critically dependent on the supporting TME [40]. Previous studies in murine models have shown that vaccination against tumor vasculature in tumor stroma, results in tumor repression without significant adverse effects, suggesting that TME-targeted immunotherapy is likely to bring a benefit to cancer patients [41-43].

However, tumor immune tolerance is a major impediment in cancer immunotherapy. For example, tumor vaccines proven to have therapeutic effects *in vitro* have the ability to activate the host immune system. Even the use of tumor-specific antibodies and activation of antitumor immune cells does not alter the overall capabilities of these agents [44]. Therefore, researchers began to take a fresh look at the relationship between the tumor and the TME, and determined that the failure of these vaccines is probably due to the existence of special cells in the TME that are immune-suppressive. “Antitumor” immune cells include cytotoxic CD8+ T lymphocytes (CTLs), T helper type 1 (Th1) cells, type 1 macrophages (M1), type 1 neutrophils (N1), natural killer (NK) cells, natural killer T (NK-T) cells, eosinophils, and mature dendritic cells (DCs) [45-48], all of which are known to support the clearance of tumor cells. An effective antitumor immune response can be divided into three steps: First, there is full activation of T lymphocytes by mature DCs in the tumor-draining lymph node; then, cancer-specific effector T cells leave the blood vessels and enter the tumor site; and finally, tumor-infiltrating lymphocytes (TIL) eventually cause tumor regression [49].

In contrast to normal tissues, the vast majority of immune cells in the TME have lost their function. Furthermore, in cancer patients the composition of immune cells undergoes a change wherein the inhibitory subgroups, such as regulatory immune cells, myeloid-derived suppressor cells (MDSCs), and M2 macrophages are the dominate components [50]. In addition, the TME helps the tumor cells to escape from the attack of effector cells by recruiting inhibitory cells, therefore escaping the body's immune surveillance, moreover, the origin of MDSCs in the TME is converted by a specific mature subset of NK T-cells [51]. Furthermore, the abnormal distribution, migration barrier, and anergy of T lymphocytes and other immune cells are important reasons for T lymphocyte-mediated antitumor activity failure [52-54]. Thus, immunosuppression in the TME leads to inefficient or ineffective cancer treatments, and immunotherapy strategies aimed at activating T cells are currently under investigation in preclinical and clinical studies.

FAP α is an immune-suppressive component in the TME. The first experiments that assessed the immunologic effects of perturbing the FAP α+ stromal cells used a direct approach of conditionally depleting this cell type from a mouse bearing immunogenic Lewis lung carcinoma cells expressing ovalbumin (LL2/OVA). These studies demonstrated that administering diphtheria toxin to these mice depletes approximately 80% of the tumoral FAP α+ cells, which comprised only approximately 2% of all tumoral cells, and caused rapid, adaptive immune-dependent reduction in tumor volume. Furthermore, when FAP α expression was inhibited, tumor shrinkage was seen accompanied by increased expression of IFN-γ and TNF-α, indicating the existence of immune suppression that was restricted to the FAP α+ cells [8]. However, the mechanism of FAP α action on immunosuppression has not been clarified in detail. In subcutaneous tumors established with immunogenic LL2/OVA, the FAP α+ population is comprised of CD45+ and CD45- cells, and the tumoral FAP α+/CD45+ population was identified as a minor sub-population of F4/80hi/CCR2+/CD206+ M2 macrophages [55]. Using bone marrow chimeric mice in which the primate diphtheria toxin receptor (DTR) is restricted either to the FAP α+/CD45+ or to the FAP α+/CD45- subset, it was demonstrated by conditionally depleting each subset that both independently contribute to the immune suppressive properties of the TME. The immune inhibitory enzyme, heme oxygenase-1 (HO-1), is the basis for the function of the FAP α+/CD45+ subset. FAP α+/CD45+ cells are the major tumoral source of HO-1, and an inhibitor of HO-1, Sn-mesoporphyrin, causes the same extent of immune-dependent arrest of LL2/OVA tumor growth as does the depletion of these cells. Since this observation of immune suppression by the FAP α+/CD45+ stromal cell has been replicated in a transplanted model of pancreatic ductal adenocarcinoma, tumoral immune suppression is likely mediated by macrophages expressing FAP α and HO-1.

In subsequent studies, immune suppression by the FAP α+ CAFs was mediated by CXCL12, the chemokine that binds to cancer cells and excludes T cells by a mechanism that depends on signaling by the CXCL12 receptor, CXCR4 [56]. T cells are absent from regions of the tumor containing cancer cells, which are coated with the chemokine, CXCL12, and the FAP α+ CAFs are the principal source of CXCL12 in the tumor. Administering AMD3100, a CXCL12 receptor chemokine (C-X-C motif) receptor 4 inhibitor, induces rapid T-cell accumulation and acts synergistically with α-PD-L1 to greatly diminish cancer cells, which are identified by their loss of heterozygosity of the *TRP53* gene. The residual tumor is composed only of premalignant epithelial cells and inflammatory cells. Thus, a single protein, CXCL12, secreted from a single stromal cell type, the FAP α+ CAFs, explains the overriding immunosuppression by the FAP α+ cell in a model of human pancreatic ductal adenocarcinoma. CXCL12 is the reason for immune suppression, while abrogation of FAP α positive cells permits immune inhibition of tumor growth and enhances the efficacy of constructed immunotherapeutic antibodies [57]. Furthermore, a newly developed vaccine that co-targets tumor cells and FAP α, a consistent marker of CAFs, have shown greater antitumor activity with the enhanced induction of infiltration of CD8+ T cells in B16 melanoma models [58]. Therefore, the more we can understand about the role of FAP α in the suppression of the antitumor response of the immune system will lead to novel immunotherapy targets for clinical benefit.

## THE FAP α-TARGETED IMMUNOTHERAPY STRATEGY

As previously mentioned, FAP α is a transmembrane serine protease that is highly expressed on CAFs present in >90% of human epithelial tumors, and plays a significant role in tumor progression and metastasis [14]. Targeting FAP α genetically with vaccines, with antibodies, or with pharmacological agents, impairs tumor progression in several preclinical cancer models [59-61]. Therefore, FAP α is considered to be an adaptive tumor-associated antigen for tumor immunotherapy. In this section we will focus on the clinical and preclinical attempts at employing FAP α-targeted treatment strategies and its prospects in tumor therapy.

As reviewed above, FAP α belongs to the serine proteinase family, has both collagenase and dipeptidase activities which can degrade gelatin, collagen, and other substrates of dipeptidase, and promotes tumor growth, migration, invasion, metastasis and ECM degradation. Therefore, it was hypothesized that selectively blocking the enzymatic activity of FAP α may be a method of targeting it in tumor development. Val-boroPro, also called Talabostat, was the first inhibitor of the proteolytic activity of FAP α used in phase II clinical trials successively performed in patients with metastatic colon cancer, non-small cell lung cancer, and melanoma. Val-boroPro was administrated alone or combined with other non-specific anti-tumor drugs, but minimal clinical response was observed with the addition of Val-boroPro [62-64]. There is still no consistent conclusion as to why the drug did not work, but there are reports suggesting that the enzymatic activity of FAP α has little to do with increased tumor growth [28], and there are also contradictory results suggesting that serine proteases can function as tumor suppressors [21, 65]. Some scientists hold the view that it was the form that Val-boroPro exists *in vivo* that matters [4], and that more studies are required to get the real picture.

The disappointing clinical outcome of Val-boroPro does not exclude the potential role of FAP α's proteolytic activity in tumor invasion and metastasis, or that an inhibitor antibody may be a potent therapeutic target. An inhibitory scFv antibody, named E3 was identified, which competitively inhibits FAP α function [66]. This scFv antibody with high affinity and enhanced inhibitory effects on FAP α enzyme activity, seems very likely to be exploited as a tool for the treatment of FAP α driven tumors. Studies to evaluate the effects of scFv antibody deserve more exploration and further characterization to confirm previous findings. In one study, human scFvs were transformed into bivalent minibodies of completely human origin, which worked far better than murine or humanized antibody derivatives. Thus, the successful use of mini-antibodies in immunohistology for a variety of carcinomas is encouraging for *in vivo* diagnostic and tumor-targeting studies [67]. Combinatorial strategies addressing the two key issues of cancer immunotherapy (ie. targeting the tumor cells and modulating the T-cell response), the production of a bio-specific single chain antibody (scFv) directed against FAP α and CD3 (T cell receptor component), and the subsequent construction of a bio-specific antibody combined with co-stimulatory antibody-ligand fusion proteins, show the potential for initiating and regulating immune response at the TME in addition to modulating tumor progress [68, 69]. Advanced combinatorial strategies could result in unprecedented clinical outcomes with great beneficial effects.

Early work on FAP α-targeting monoclonal antibodies investigated the toxicity, imaging, and bio-distribution of a 131-labeled monoclonal antibody (131I-mAbF19) against FAP α in patients with hepatic metastases from colorectal carcinoma. The 131I-mAbF19 was administered by intravenous injection and no toxicity was observed [70]. Further work has defined the population pharmacokinetics of 131I-mAbF19 with the data from two phase I studies in cancer patients [71]. These studies indicated potential diagnostic and therapeutic applications of humanized mAbF19. Then, sibrotuzumab, an antibody directed against humanized F19, was produced and used in a Phase I dose-escalation study, which showed the safety of this antibody in patients who suffer from advanced or metastatic FAP α-positive cancers [6]. Interestingly, there was a relationship between body weight and this antibody [72]. Unconjugated sibrotuzumab (BIBH 1) was investigated in an early phase II trial with metastatic colorectal cancer patients, and, although it was well tolerated and safe, the trial was suspended because of its minimal clinical response [73]. Despite the disappointing results, the study of more efficient FAP α antibodies continues. Novel human-mouse cross-reactive antibodies, ESC11 and ESC14 labeled with radionuclide (177) Lu, were recently engineered and characterized [74]. Accumulation of these two antibodies is specific to tumor tissue, particularly the (177) Lu-labeled ESC11, suggesting these antibodies could be potential tumor growth retardants in a melanoma xenograft model. (177)Lu-ESC11 has advantage over (177) Lu-ESC14 and (177) Lu-vF19 in prolonging survival time. However, more preclinical and clinical experiments are needed to explore the diagnostic and therapeutic effects of these potent antibody-drug conjugates in patients with FAP α-expressing tumors.

Vaccines targeting FAP α provide another therapeutic strategy that takes advantage of the restricted distribution of FAP α in tumor sites. Scientists have constructed a DNA vaccine directed against FAP α [75], and immune tolerance against the FAP α self-antigen can be inhibited through delivery of FAP α cDNA as a subcutaneous DNA vaccine. In prophylactic experiments, the CD8+ T-cell-mediated antitumor immune response induced by pFAP α vaccination inhibited tumor growth, significantly suppressed growth of pulmonary metastases and prolonged the life spans of vaccinated mice, consistent with a previous study [60]. Similar results were also observed for adaptive immunity induced by adoptive transfer of T cells from pFAP α-immunized mice. Non-specific immune responses were unlikely, because the cytotoxic effects mediated by CD8+ T cells *in vitro* were restricted to target cells overexpressing the FAP α antigen, consistent with other studies [59, 60]. More importantly, an *in vitro* screening method was used to determine whether dendritic cells transfected with mRNA encoding products of FAP α are capable of stimulating cytotoxic CD8+ (CTL) responses from human peripheral blood mononuclear cells. It was demonstrated that CTL responses could be consistently generated against FAP α. To enhance the immunogenicity of the mRNA-translated FAP α product, a lysosomal targeting signal derived from lysosome-associated membrane protein-1 (LAMP-1) was fused to the COOH terminus of FAP α to redirect the translated product into the class II presentation pathway. Dendritic cells transfected with mRNA encoding the FAP α-LAMP fusion product stimulated enhanced CD4+ and CD8+ T-cell responses [76].

Furthermore, in our research, we have developed a new tumor vaccine, FAP α τ-MT, which was produced by conjugating 1-MT to a FAP α. The *in vitro* results confirmed that 1-MT could be dissociated from the FAP α τ-MT vaccine and inhibit intracellular IDO activity [77]. In a FAP α-positive tumor model, the FAP ατ-MT vaccine elicited an antitumor response that was similar to systemic treatment with the FAP α τ vaccine plus 1-MT. Most importantly, administration of the FAP α τ-MT vaccine did not lead to pregnancy failure in mice carrying allogeneic fetuses. These findings that FAP α τ-MT breaks tumor immune tolerance as a local IDO inhibitor, suggesting that conjugation of 1-MT to a tumor antigen peptide is a potentially effective clinical cancer immunotherapy. In subsequent studies, we used the main catalytic domain of dipeptidyl peptidase of murine FAP α as a vaccine that contains abundant T-cell epitopes and B-cell epitopes, combined with curcumin lavage that inhibits the expression of IDO to relieve tumor immune tolerance, to treat mice implanted with melanoma cells. We demonstrated that FAP α vaccine combined with curcumin lavage inhibits tumor growth and prolongs the survival of mice implanted with melanoma cells. The combination of a FAP α vaccine and curcumin stimulated FAP α antibody production and CD8+T cell-mediated killing of FAP α-expressing stromal cells without adverse reactive effects [9]. These results suggest that FAP α, a product preferentially expressed by CAFs, would be a more effective antigen to target in the setting of cancer immunotherapy.

## CONCLUSIONS

Cancer cells are embedded in stroma, the connective tissue framework of solid tumors. Stromal cells and cancer cells depend on each other for mutual paracrine stimulation, and stromal fibroblasts are probably required for cancer cells to survive and grow [78, 79]. Recently, tumor stromal cells have been suggested as a target for tumor immunotherapy because tumor stromal cells, unlike tumor cells, are diploid, genetically stable, and open to immunological attack. Thus, immunizing against stromal fibroblasts in tumors may unmask an immune response to cancer [80-82].

FAP α is a tumor-associated antigen which is a serine protease involved in extracellular matrix remodeling and highly expressed on reactive stromal fibroblasts in >90% of human epithelial carcinomas, but is not detectable in normal adult human tissues [83, 84]. Stromal cells expressing FAP α may suppress the immune response to tumors as a consequence of producing massive amounts of stromal cell-derived factor-1 (SDF-1/CXCL12). SDF-1 attracts regulatory T cells (CD4+ subtype) into the tumor [85]. Furthermore, because of the multiple roles played by FAP α in neoangiogenesis, invasion and metastasis, it is being explored as a target for cancer therapy.

**Figure 2:**
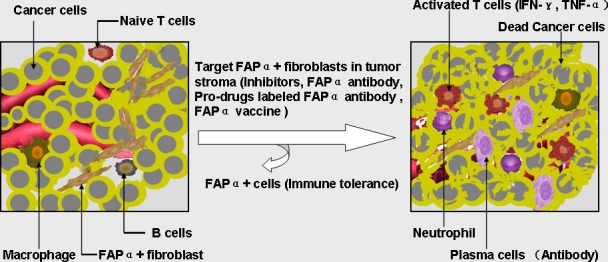
The diagram demonstrating the role of FAP α in immune suppression and the application of FAP α-targeted immunotherapy strategy

In the context of immunotherapy involving T cells targeting cancer cells, an agent targeting FAP α-expressing cells might increase therapeutic efficacy against both solid tumors and metastatic cells [86-89]. Eliminating FAP α+ stromal fibroblasts activating cancer-specific T cells should inhibit growth of small spontaneous tumors and thus may help eliminate clinically undetectable cancer cells that have already metastasized before excision of the primary tumor [90]. Thus, combined immunotherapy treatment consisting of T cells that target cancer cells and an agent targeting FAP α-expressing cells for destruction could increase the success of eliminating solid tumors and metastatic cells. Because FAP α is a robust target for immunotherapy, preclinical and clinical studies targeting FAP α have been initiated adopting methods such as small inhibitor molecules, FAP α-activated prodrugs, monoantibodies, DNA vaccines, peptide vaccines, and modified vaccines, etc. (Figure 2). In the future, there will be more and more combinatorial immuno(chemo) therapeutic regimens and other promising methods targeting FAP α.
